# Evaluating sensitivity and specificity of handheld point-of-care ultrasound testing for gynecologic pathology: a pilot study for use in low resource settings

**DOI:** 10.1186/s12880-020-00518-8

**Published:** 2020-10-27

**Authors:** Marika Toscano, Kam Szlachetka, Natalie Whaley, Loralei L. Thornburg

**Affiliations:** Department of Obstetrics and Gynecology, University of Rochester Medical Center, 601 Elmwood Ave, Box 668, Rochester, NY 14642 USA

**Keywords:** Point-of-care ultrasound, POCUS, Low-resource setting, Pelvic ultrasound, Sensitivity, Specificity

## Abstract

**Background:**

Point-of-care ultrasound (POC-US) is a diagnostic test conducted at the site of patient care with direct interpretation by the clinician, providing immediate results. POC-US for gynecologic application is not well characterized by current literature yet has the potential to increase access in limited resource settings. We compared the diagnostics of three POC-US devices for gynecologic (GYN) pathology and then performed evaluation of sensitivity and specificity of a single best POC-US device for intended use in a low resource setting.

**Methods:**

This is prospective, pilot descriptive study of 60 subjects. In part 1, comparison of three POC-US devices was performed. Twenty subjects underwent POC-US with three test units [GE Vscan (Vscan), Sonosite Iviz (Iviz), Philips Lumify (Lumify)] followed by diagnostic ultrasound (Dx-US) for reference imaging. Image quality and correlation for devices was scored by blinded reviewers and quantitative measurements of GYN pathology were compared. In part 2, forty subjects underwent POC-US validation with the highest scoring device (Lumify) and Dx-US for reference imaging. Concordance of POC-US operator-interpreted diagnosis with reference imaging interpretation were assessed by Cohen’s unweighted kappa coefficient. Accuracy and agreement of POC-US were assessed by linear regression and Bland–Altman plot analysis. Sensitivity and specificity of POC-US for gynecologic pathologies were calculated.

**Results:**

In aggregate qualitative measurements, Lumify and Iviz units performed superiorly to Vscan. There was no statistically significant difference in quantitative measurements between devices, but a trend towards lower mean error was seen for Lumify and Iviz as compared to Vscan. Lumify device had highest overall scoring and was selected for further testing. In validation comparison of Lumify to Dx-US, no statistically significant differences were found for measurements of endometrium, uterus, ovaries, adnexal pathology, or leiomyomata, (*P* < 0.02) with excellent agreement in operator-interpreted diagnosis (Kappa > 0.7). Sensitivity and specificity of detecting pathology was 80–100% with PPV and NPV 76–100%.

**Conclusion:**

Among three POC-US devices, Lumify and Iviz devices show highest potential for successful application to clinical gynecologic ultrasound. Clinician-performed POC-US has high diagnostic accuracy, sensitivity, and specificity for basic GYN anatomy and pathology. POC-US is an acceptable and feasible diagnostic tool with potential for future application in a low resource setting to increase access to ultrasound.

## Background

Point of care ultrasound testing (POC-US) is an ultrasound performed at the bedside by the clinician and interpreted directly by the clinician for immediate diagnosis and treatment planning [1]. Ultrasound is cost-effective, safe, and has the benefit of real-time imaging for immediate diagnosis. However, it is highly operator and equipment dependent which carries implications about its use in guiding moment-to-moment therapeutic decisions when used in a point-of-care context.

Numerous leading ultrasound-manufacturing companies have developed lightweight handheld POC-US devices, including smartphone-based devices. Handheld POC-US devices allow physicians greater ease of use in a variety of settings, including low resource international and rural settings. POC-US devices have an array of available software and probes and sources report good agreement in image quality between these devices and higher-end machines [2–6], though some reports demonstrate lower resolution and total image quality which could lead to missed diagnoses [5].

As a result of its ease of use and portability, POC-US has the potential to be very influential in low-resource settings, particularly in international medical work in low- and middle-income countries [7]. In this setting, patients often must travel long distances to access medical care and may not be able to afford these travel and health care costs. POC-US testing can more easily triage patients who are high risk or require a higher level of care. The literature supports this hypothesis. Numerous studies demonstrate that POC-US applied broadly over multiple organ systems in a variety of low resource settings adds to clinical diagnosis and influences outcomes and decision making regarding treatment plan [8–12].

The representation of gynecologic ultrasound in these studies is very limited [13–15]. Two studies utilized a portable device [14, 15], but none used handheld devices or studied gynecologists as ultrasound operators. Therefore, the aim of this study was to investigate the utility of gynecologist-performed handheld POC-US to detect gynecologic pathology as a pilot for future application of use in a low resource setting to triage patients with a gynecologic complaints to a higher level of care. This study intended to investigate gynecologic-ultrasound trained practitioners as ultrasound operators.

In this study, three handheld POC-US devices were compared for use in clinician-performed imaging and diagnosis of common gynecologic pathologies in order to select the device best suited for use in a future study in a low-resource setting. The selected device then underwent further validation testing for diagnostic accuracy. The primary outcome of this study was to evaluate sensitivity and specificity of gynecologist-performed and interpreted POC-US for gynecologic pathology. Secondary outcomes were (1) correlation in measurements of gynecologic structures by POC-US as compared to reference imaging and (2) diagnostic concordance of operator-interpreted POC-US images as compared to interpretation of reference imaging by radiologist. The chosen POC-US was then applied to a field study to demonstrate feasibility in a low-resource setting.

## Methods

A total of three ultrasound devices were chosen for evaluation based on ability for the study team to obtain the device, prior FDA approval for gynecologic imaging, weight less than 2 lb, and cost less than $10,000, making it a reasonable option for POC-US in a low resource setting. No ultrasound company was involved in the design or analysis of the study, and did not have input into the results, scoring or publication. For details of the selected test devices, see “Appendix 1”.

Ethical approval was obtained from the University of Rochester Medical Center institutional review board (Rochester, NY), and research support was obtained from University of Rochester Department of Obstetrics and Gynecology through intradepartmental grant funding application. A total of 60 subjects were prospectively enrolled in a sequential convenience sample. Subjects were women previously scheduled by their primary gynecologist for a pelvic ultrasound for any reason (new complaint or follow-up of prior known pathology) at a University-affiliated clinic with specialization in OBGYN ultrasound. Women were enrolled sequentially from October 1 to 31, 2017 on a volunteer basis with small monetary compensation provided. Women were excluded if they were less than age 18 at the time of the study, non-English speaking, lacked legal competence to make medical decisions, were currently pregnant, or were currently incarcerated. Informed consent was obtained, and subjects underwent a transabdominal-only assessment of GYN organs and pelvis, irrespective of bladder filling, in accordance with AIUM (American Institute of Ultrasound in Medicine) Practice Parameter for the Performance of an Ultrasound Examination of the Female Pelvis [16]. Ultrasound was performed by a gynecologic-ultrasound trained practitioner as ultrasound operator (M.T.). All images were labeled according to anonymous study ID and stored on secure online database.

### Overview of study design


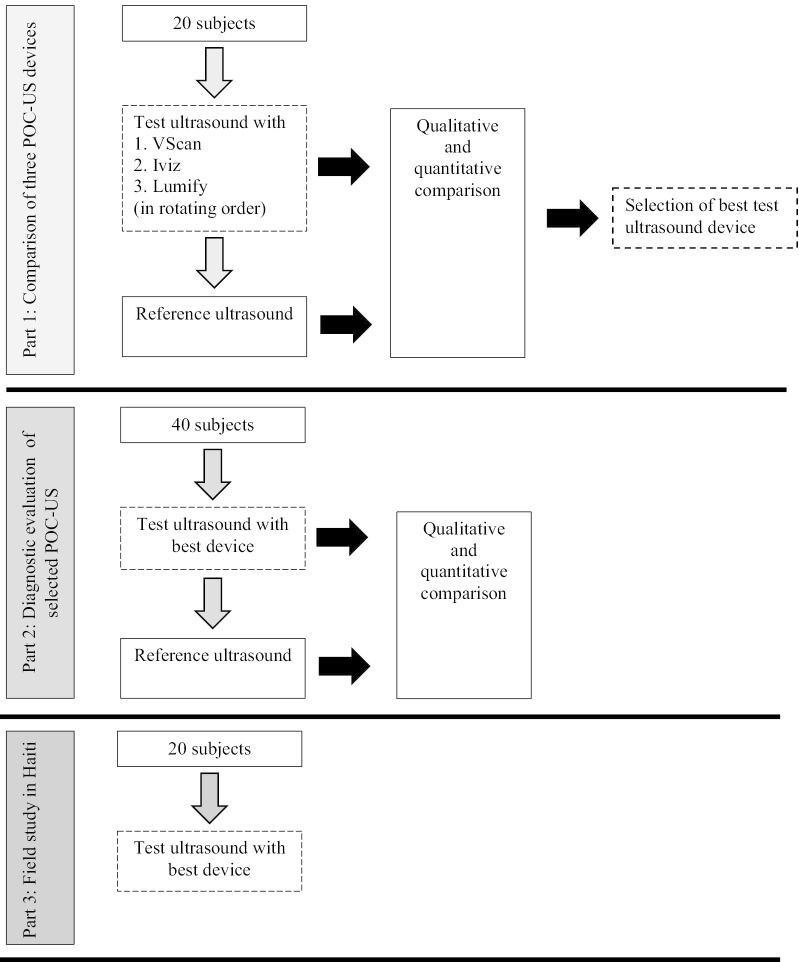


### Part 1: Comparison of three POC-US devices

Because this was a pilot study, sample size was not powered for statistical significance. The first twenty sequentially-enrolled subjects underwent clinician-performed pelvic POC-US with each of the 3 units (GE Vscan (Vscan), Sonosite Iviz + L38v linear probe (Iviz), Philips Lumify C5-2 curvilinear probe + Samsung Galaxy Tab S2 9.7 with mobile app (Lumify)) in rotating order followed by diagnostic pelvic ultrasound (Dx-US) using one of the following machines: Philips IU22 (Koninklijke Philips N.V, Amsterdam, Netherlands), Medison Accuvix 20 (Samsung Medison, Seoul, South Korea), Voluson E10 (GE Healthcare Ultrasound, Milwaukee, WI, USA) with 4–5 MHz abdominal probe. Clinician-performed POC-US was performed by single author (MT), a third-year resident in Obstetrics and Gynecology, with supervision by RDMS certified sonographer and co-investigator (KS). POC-US was performed prior to reference imaging, therefore reference test results were not available to investigators at the time of POC-US. A diagnostic ultrasound (Dx-US) in accordance with AIUM practice parameters for performance of an ultrasound examination of the female pelvis was then performed by a different OBGYN sonographer (RDMS) who was blinded to any findings on the POC-US [16].

Test images from each POC-US were blindly compared to Dx-US images by two independent sonographer reviewers. Reviewers completed a qualitative assessment for each test image of POC-US image quality and for correlation of POC-US image to Dx-US image by non-validated 5-point Likert scale from 0–5 (0—structure not seen, 1—poor to 5—excellent) for each test image. The following gynecologic structures were scored: uterus, endometrium, cervix, left ovary, right ovary, cul de sac, pathology of interest. Gynecologic structures were considered satisfactorily assessed by a POC-US device if the mean sonographer review score was ≥ 3 for image quality score and ≥ 3 for correlation score. Collapsing Likert scale response into dichotomous neutral/positive (score 3, 4 or 5) or negative (score 1, 2) categories performed to minimize ambiguity and clarify intent of responder to better capture trends in data. Neutral response aggregated with positive response to reduce response bias related to survey satisficing and maintain focus on lack of negative score as primary outcome of interest [17]. Aggregate score for each device was calculated by totaling the number of scans possessing qualitative assessment scores ≥ 3 for all structures. Two-tailed Fisher’s exact tests used to compare proportions of scans with assessment scores ≥ 3 between devices to inform superiority of a single POC-US device.

Qualitative measurements of gynecologic structures by each POC-US device were compared to measurements obtained by Dx-US for uterine volume, endometrial thickness, left and right ovary mean diameter, and pathologic structure mean diameter. For both POC-US and Dx-US, uterine volume was calculated by measuring the maximum length (excluding the cervical component), anteroposterior and transverse diameters of the uterine corpus, and using the formula for the volume of a prolate ellipsoid: V = 0.52 × (L × AP × T) [18]. Endometrial thickness was measured at the thickest part of the endometrium perpendicular to its longitudinal plane in the anteroposterior diameter from echogenic to echogenic border. Ovaries were measured in 3 dimensions (longitudinal, transverse, and anteroposterior diameters) on views obtained in 2 orthogonal planes [16]. Pathologic structures included simple or complex adnexal cysts or myomas. Simple ovarian cysts were defined as > 3 cm, thin and smooth walled, round or oval, anechoic spaces with no flow by means of color Doppler US [19]. Paraovarian and paratubal cysts were considered together with ovarian cysts. Simple ovarian cysts were measured in 3 dimensions (longitudinal, transverse, and anteroposterior diameters) on views obtained in 2 orthogonal planes. Cysts of any size which contained septations, solid or mixed cystic/solid components and were considered complex ovarian cysts and were measured as described above. Myomas were measured in three perpendicular diameters [18]. Endometrial pathology defined as thickened endometrium > 4 mm in a post-menopausal subject [20], fluid filled endometrium or IUD in situ. Endometrial measurements > 4 mm in a pre-menopausal subject were not considered pathology. Absolute mean difference in measurements defined as absolute difference in measurement by POC-US minus Dx-US. *P* < 0.05 for absolute mean difference considered significant “disagreement” in measurements and one-way analysis of variance performed to inform superiority of a single POC-US device.

A single best POC-US device was then selected by a combination of (1) highest aggregate score for image quality > 3, (2) highest aggregate score for image correlation > 3, and (3) lowest number of “disagreements” in quantitative measurements of individual structures (as defined above), and (4) suitability for field use based on total continuous scan time on one battery charge and ease of use.

### Part 2: Diagnostic evaluation of selected POC-US

The selected device then underwent further testing with additional prospective enrollment of forty subjects in a sequential convenience sample. Because this was a pilot study, sample size was not powered for significance, though a larger sample size of 40 was chosen to allow for inclusion of subjects with a variety of gynecologic pathologies, each of which has a baseline low prevalence. Clinician performed POC-US with selected device was performed by single blinded operator (MT) and Dx-US by RDMS. POC-US was again performed prior to reference imaging, therefore results of reference imaging were not available to POC-US operator at the time of image acquisition or interpretation. Similarly, results of POC-US were unknown to RDMS at the time of reference imaging. Interpretation of test images for diagnosis was performed by clinician POC-US operator (MT) at the time of image acquisition. Interpretation of reference imaging for diagnosis was by board-certified maternal–fetal medicine specialists who were blind to results of POC-US. At this research institution, maternal–fetal medicine specialists are also certified by AIUM for interpreting gynecologic ultrasound studies. All indeterminate or missing results from the POC-US were considered false-negative (in those with pathology present on reference imaging) or false-positive (in those with pathology absent on reference imaging).

### Field study procedures

A pilot field study of POC-US in a low-resource setting was performed in the remote and mountainous Borgne community on the north coast of Haiti, west of Cap Haitian. The region is accessible only by rough footpaths. The majority of the population lives in extreme poverty and relies on subsistence agriculture, fishing and trade of crops at local/regional markets. There is a low level of educational attainment and limited access to clean water or sanitation and no current access to diagnostic imaging services.

For this prospective field study, 20 total subjects were enrolled in a sequential convenience sample. Because this was a pilot study, sample size was not powered for statistical significance. Subjects were women presenting to a mobile health clinic for any complaint of possible gynecologic etiology. Prior assessment of the perceived women’s health needs in the mobile clinics revealed a need for improved general gynecologic care for benign and routine pathologies, most commonly abnormal uterine bleeding and pelvic pain. Women were approached and enrolled in January, 2018 with the help of Haitian Creole translator. Women were excluded if they were less than age 18 at the time of the study or currently pregnant. Subjects did not receive compensation or incentives for participation. Clinician performed POC-US was performed and interpreted by single author (MT) with supervision by co-investigator (NW) after patient consent.

Because there is no access to imaging capabilities in the region of the field study, no reference imaging was performed for comparison to POC-US imaging for subjects in the field study.

### Statistical analysis

Demographic characteristics of the two groups were compared with chi square tests for categorical variables. Distribution of continuous demographic data was established using the Shapiro–Wilk test followed by Mann–Whitney U test for non-parametric data.

Two-tailed Fisher’s exact tests were used to compare proportions of scans with assessment scores ≥ 3 between the three test devices to inform superiority of a single POC-US device. Absolute mean difference in measurements of gynecologic structures (defined as absolute difference in measurement by POC-US minus Dx-US) compared with one-way analysis of variance to inform superiority of a single POC-US device.

Concordance of diagnoses between POC-US and Dx-US was assessed with Cohen’s unweighted kappa coefficient with *P* value < 0.05 defining agreement of nominal variables exceeding that expected under the null hypothesis. For analysis, subjects with more than one diagnosis were included in multiple categories.

Continuous variables compared by linear regression with a value close to 1 representing high agreement between device measurements. Bland–Altman plots were constructed as a visual representation of the mean difference between single paired measurements by the two methods. The limits of agreement indicated by the dotted lines and calculated as the interval of two standard deviations of the measurement differences on either side of the mean difference (solid line). A priori determination of acceptable limits of agreement was not established. Agreement in measurements defined as 95% of data points (difference between the two measurements) included in the 95% limits of agreement. Sensitivity, specificity, positive predictive value and negative predictive value were also calculated by 2 × 2 contingency table for 1) dichotomous outcome of presence or absence of pathology and 2) stratified by individual diagnoses. All statistical analyses were performed using IBM SPSS Statistics 21.

## Results

This protocol utilized the STARD 2015 standard for reporting diagnostic accuracy studies [21].

**Fig. 1:**
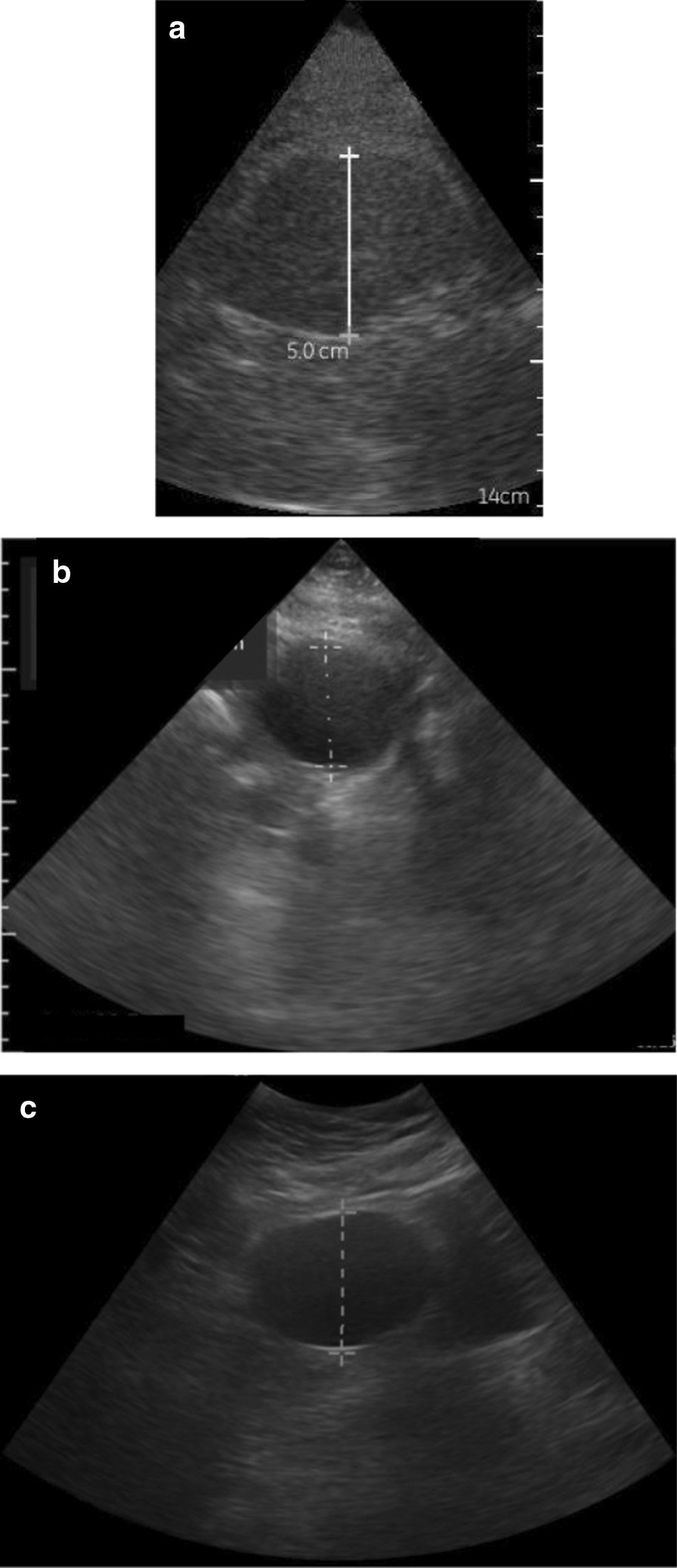
Comparative imaging of simple ovarian cyst with **a** GE Vscan, **b** Sonosite Iviz, **c** Philips Lumify

Of 60 total participants in the United States, the majority of subjects were Caucasian, overweight/obese and parous. Abnormal uterine bleeding and ovarian cyst were the most common indications for pelvic ultrasound (Table 1). Representative images from each of the three POC-US test devices are shown for an ovarian cyst (Fig. 1). No adverse events occurred secondary to POC-US units or reference imaging during this study.

**Table 1:** Baseline characteristics of study participants

Characteristic	United States study participants (n = 60)	Field study participants (n = 20)	*P *value
Age^a^ (years)	36 (28.5–38)	31 (24–53.5)	0.56^c^
BMI > 30	23/60 (38%)	0/20 (0%)	< 0.01
Parity^a^	1 (0–2)	2 (0.5–5.5)	0.13^c^
Race			< 0.01
Caucasian	40/60 (67%)	0 (0%)	
Black	17/60 (28%)	20/20 (100%)	
Other	3/60 (5%)	0 (0%)	
Ethnic origin			0.23^d^
Hispanic	5/60 (8%)	0/20 (0%)	
Non-Hispanic	55/60 (92%)	20/20 (100%)	
Menopausal status			0.64^d^
Pre-menopausal	48/60 (80%)	16/20 (80%)	
Post-menopausal	12/60 (20%)	4/20 (20%)	
Day of menstrual cycle^b^	20 ± 12	Unknown	n/a
Indication for USN			< 0.01
Pelvic Pain	10/60 (17%)	20/20 (100%)	
Abnormal uterine bleeding	17/60 (28%)	0/20 (0%)	
IUD localization	8/60 (13%)	0/20 (0%)	
Ovarian cyst	16/60 (27%)	0/20 (0%)	
Follow up of known pathology	9/60 (15%)	0/20 (0%)	

All data presented as n(%) unless otherwise noted

*n/a* not applicable

All *p *values calculated with chi-square test unless otherwise noted

^a^Median (interquartile range)

^b^Mean ± standard deviation

^c^Mann–Whitney U test used to calculate *p *value

^d^Fisher’s exact test used to calculate *p *value

### Part 1: Comparison of three POC-US devices

Of the 20 total participants who underwent POC-US assessment for device comparison, reference imaging demonstrated 4 subjects with myomas, 8 subjects with adnexal pathology (2 complex and 6 simple ovarian cysts), and 6 subjects with endometrial pathology (4 with IUD in situ, 2 with thickened endometrium).

**Fig. 2:**
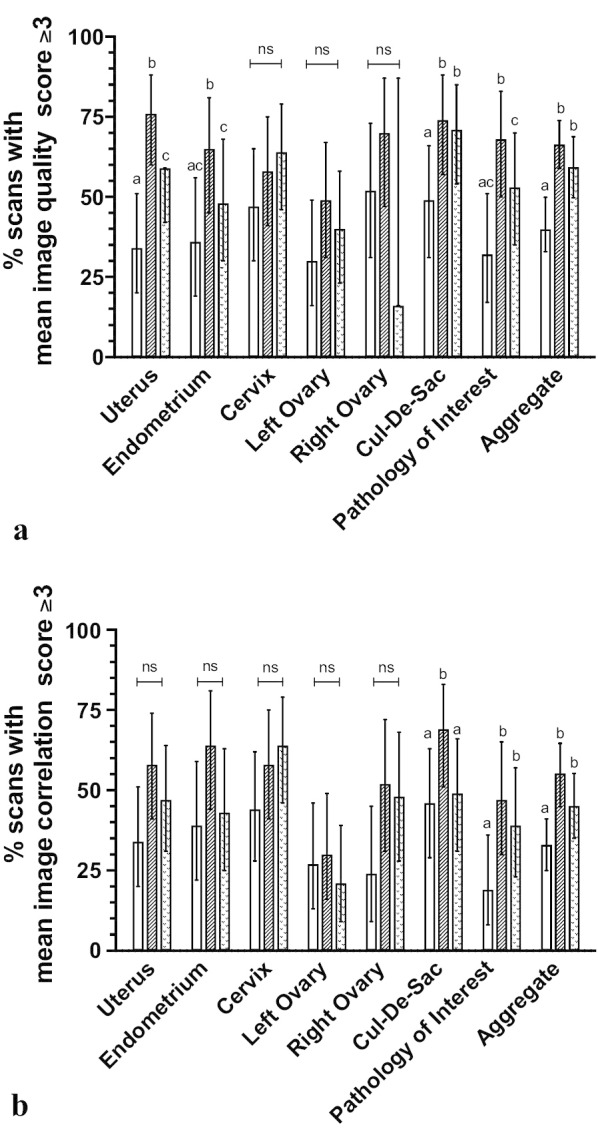
Bar graphs comparing, **a** image quality score, **b** correlation to diagnostic ultrasound image score for three POC-US devices. Bars followed by a different letter are significantly different at alpha level of 0.05 by Fisher’s exact test. Error bars represent 95% CI

Satisfactory assessment of gynecologic structure as defined by mean image quality score ≥ 3/5 and correlation score ≥ 3/5 occurred most frequently using Iviz, then Lumify and then Vscan (Fig. 2).

Image quality scores did not statistically significantly differ between devices for cervix, right ovary or left ovary. There were statistically significant differences between POC-US devices for uterus, endometrium, cul-de-sac, pathology of interest, and aggregate score (Fig. 2a). The Iviz and Lumify had statistically higher number of scans with image quality scored ≥ 3/5 as compared to the Vscan for uterus, cul-de-sac, pathology of interest, and aggregate image quality. The Iviz had statistically higher number of scans with image quality scored ≥ 3/5 as compared to the Lumify for uterus, endometrium, and pathology of interest (Fig. 2a). Compared to the Vscan, Iviz and Lumify were both statistically significantly more likely to have an aggregate image quality score ≥ 3/5 (66% Iviz versus 39% Vscan, *P* < 0.01; 59% Lumify versus 39% Vscan, *P* < 0.01) with no significant difference between Iviz and Lumify.

Image correlation scores did not statistically significantly differ between devices for uterus, endometrium, cervix, left ovary and right ovary. There were statistically significant differences between groups for image correlation score for cul-de-sac, pathology of interest, and aggregate (Fig. 2b). The Iviz device had a statistically significantly higher aggregate number of scans with image correlation scored ≥ 3/5 as compared to Lumify or Vscan (33% Vscan vs. 55% Iviz, *P* < 0.01; 45% Lumify vs. 55% Iviz, *P* < 0.01), with no difference between Vscan and Lumify.

**Fig. 3:**
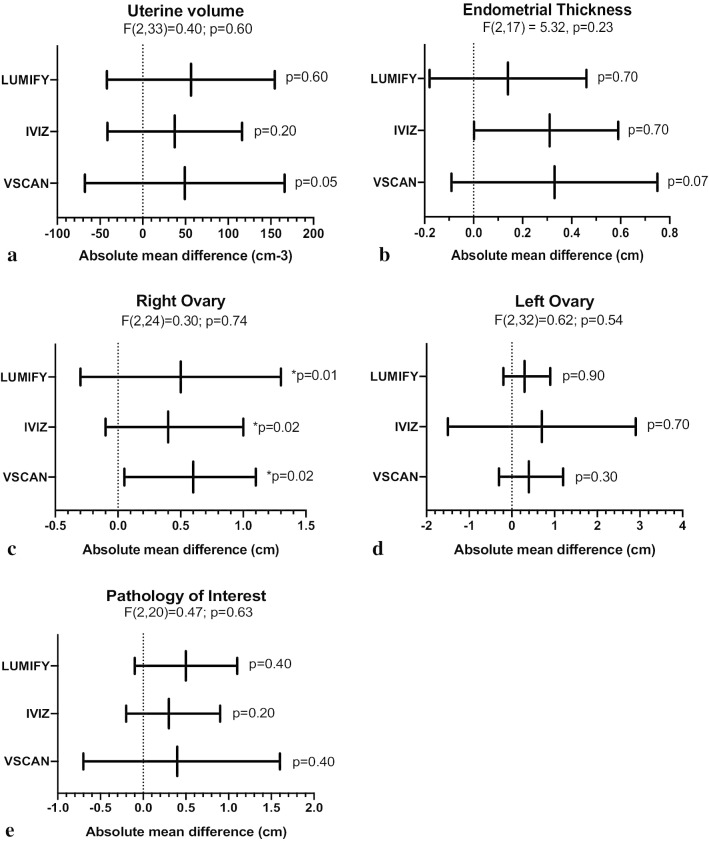
Agreement between measurements of three POC-US devices and diagnostic ultrasound. Absolute mean difference in measurements for **a** uterine volume, **b** endometrial thickness, **c** right ovarian mean diameter, **d** left ovarian mean diameter, **e** pathology of interest mean diameter shown with error bars representing 95% limits of agreement. Bars followed by asterisk indicate a significant difference in measurements between POC-US and Dx-US by *t *test at alpha level of 0.05

On quantitative device comparison, the absolute mean difference in measurements between diagnostic ultrasound and each of three devices did not have statistically significant disagreement for endometrial thickness, uterine volume, left ovarian diameter and pathologic structure mean diameter (Fig. 3). The opposite finding was demonstrated for measurements of right mean ovarian diameter which had significant disagreement in measurements by POC-US as compared to Dx-Us (Fig. 3). There were no statistically significant differences between devices for any measurement as determined by one-way analysis of variance (Fig. 3).

Based on these data, all three devices performed equivalently on quantitative device comparison, though there was trend towards decreased mean error in measurements and narrower limits of agreement for both Iviz and Lumify as compared to Vscan. The Iviz and Lumify performed overall equivalently and Vscan device inferiorly in qualitative measurements. Ultimately, Lumify was selected based on superior overall ease of use and applicability to low-resource setting in regarding to battery life, portability, and cost. In addition, Lumify was found to be superior in ability to adjust depth and gain.

### Part 2: Diagnostic evaluation of selected POC-US

Between October 1 and 31, 2017, 40 patients were assessed for initial eligibility and invited to participate. “Appendix 2” shows the flow of patients through the study, along with the primary outcome of gynecologist performed and interpreted POC-US of gynecologic pathology. Patients who were excluded (and reasons for this) or who withdrew from the study are noted. In total, 40 patients completed the study, a completion rate of 100%. Subjects with more than one diagnosis were included in multiple categories therefore total number of included test subjects was 110.

Of 40 total participants who underwent POC-US with Lumify, reference imaging showed 10 subjects with myomas (mean diameter 3.9 cm), 5 subjects with adnexal pathology [4 with simple cysts (mean diameter 4.9 cm) and 1 hemorrhagic cyst (mean diameter 2.3 cm)], 12 subjects with physiologic follicular cysts (mean diameter 1.7 cm) and 10 subjects with endometrial pathology [6 subjects with IUD in situ, 3 subjects with post-menopausal thickened endometrium (mean thickness 0.7 cm)].

In validation phase, the Lumify had a high overall statistical concordance with Dx-US in operator-interpreted diagnosis of normal pelvic anatomic structures as well as pelvic pathology as assessed by Cohen’s unweighted kappa (Table 2). There was almost perfect agreement in assessing presence of endometrial pathology and type of endometrial pathology (κ = 0.940, 0.841, respectively *P* < 0.01). There was substantial agreement in assessing uterine position, presence of fibroids, presence of adnexal pathology, type of adnexal pathology, and final diagnosis (κ = 0.752, 0.731, 0.701, 0.716, 0.761 respectively, *P* < 0.01). There was fair agreement between in ability to assess right ovary and left ovary (κ = 0.357, 0.415, *P* = 0.022 and *P* < 0.01 respectively).

**Table 2:** Concordance of nominal variables Lumify POC-US versus Dx-Us

Variable	n^a^	Categories	Cohen’s unweighted kappa coefficient	*p* value
Uterine position	43	Anteverted, retroverted, mid-position	0.7	< 0.0001
Endometrial pathology	37	Yes, no	0.9	< 0.0001
Endometrial pathology type	12	Thickened, fluid, IUD, none	0.8	< 0.0001
Fibroids	39	Yes, no	0.7	< 0.0001
Right ovary	38	Seen, not seen	0.4	0.02
Left ovary	37	Seen, not seen	0.4	0.002
Adnexal pathology present	40	Seen, not seen	0.7	< 0.0001
Side of ovarian pathology	14	Left, right	1	< 0.0001
Type of ovarian pathology	18	Normal, functional follicle, abnormal	0.7	< 0.0001
Final diagnosis^a^	50	Normal, ovarian finding, endometrial finding, fibroid, cul de sac finding	0.7	< 0.0001

^a^Subjects with more than 1 diagnosis included for each category

**Fig. 4:**
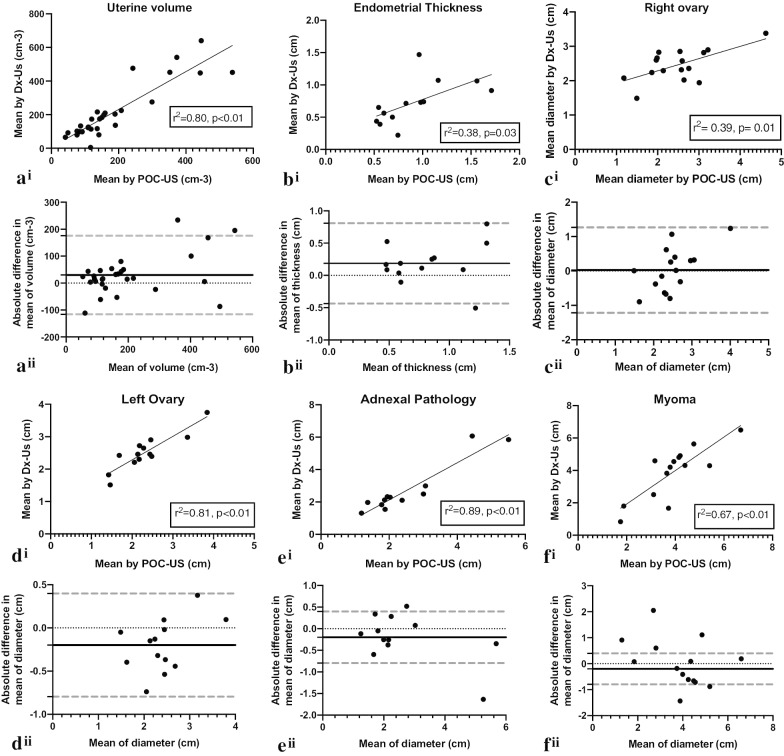
Agreement between measurements of Lumify POC-US and Dx-US of **a** uterine volume, **b** endometrial thickness, **c** right ovarian mean diameter, **d** left ovarian mean diameter, **e** adnexal pathology mean diameter, **f** myoma mean diameter by (i) linear regression and (ii) Bland–Altman plot analysis.

Lumify measurements of all continuous variables (endometrial thickness, uterine volume, left and right ovarian mean diameter, adnexal pathology mean diameter and myoma mean diameter) showed no statistical disagreement from Dx-Us by linear regression (Fig. 4i). Bland–Altman plots were constructed with > 95% of data points falling within the 95% limits of agreement. Bland–Altman bias ranged 0.29 to 31.5 for these measurements (Fig. 4ii). Overall the sensitivity, specificity, positive and negative predictive value of the Lumify POCUS was excellent with a specificity and sensitivity 80–100% for detecting myoma, endometrial and adnexal pathologies, and with PPV and NPV ranging 76–100% (Table 3). Contingency tables for accuracy of POC-US are available in Additional file 1: Table S1.

**Table 3:** Sensitivity, specificity, positive predictive value and negative predictive value of Lumify POC-US for pelvic pathology

Variable	PPV (%)	NPV (%)	Sensitivity (%)	Specificity (%)
Endometrial pathology	90.9	100	100	96.4
Fibroids	80	93.1	80	93.1
Adnexal pathology	76.5	82.6	76.5	82.6
Overall	81.3	80.4	74.7	85.7

### Field study results

Demographic characteristics of subjects are shown in Table 1. As compared to subjects in the United States, those in the field study were majority Black race (100% vs. 28%, *P* < 0.01) and not overweight or obese (100% vs. 38%, *P* < 0.01). Pain was the single given indication for pelvic ultrasound in this population (100% vs. 17%, *P* < 0.01). Of 20 total participants, the POC-US identified normal gynecologic anatomy in 90% of subjects, although ability to identify ovaries was limited by transabdominal approach. Only 1 subject was identified as having leiomyomata and 1 subject had an ovarian follicular cyst.

## Discussion

Our study supports the accuracy and feasibility of gynecologist-performed and interpreted handheld point-of-care pelvic ultrasound for observing normal gynecologic anatomic structures and common pathology including fibroids, adnexal masses, and endometrial abnormalities.

In comparing three POC-US devices to diagnostic ultrasound, we demonstrated that POC-US by a clinician has acceptable image quality and correlation to reference imaging for gynecologic application. Consistent with prior studies of handheld POC-US devices applied to broader clinical contexts including emergency medicine, cardiology and internal medicine, the devices tested here had satisfactory performance compared to reference imaging [1]. Our results show superiority of the Iviz and Lumify devices in comparison to Vscan device. This comparison testing was limited to a small sample size, however the results provided sufficient information to guide our selection of a single best device and may help inform future studies of these devices for gynecologic application.

In validation testing of the Lumify device, the POC-US was able to provide comparable information to the reference imaging with high level of accuracy when used to perform a standard pelvic ultrasound. We identified a single study investigating handheld POC-US by gynecologists for gynecologic application in the literature and their reported agreement between POC-US and Dx-US was similar to ours. Sayasneh et al. studied the Vscan POC-US in a high resource setting with a comparable sample size [3]. These authors demonstrated strong agreement for final diagnosis and ovarian pathology for Vscan, where we report similar agreement for Lumify. Both of the studies also found good concordance for fibroids, but the Vscan had poor concordance for ovarian pathology whereas we found substantial agreement [3]. Our only difficulty was in assessing normal adnexa. There were statistically significant differences in measurements of non-pathologic right ovary during device comparison and authors suspect this to be a result of limitations of transabdominal approach-which is highly affected by abdominal adiposity and bladder filling. These handheld ultrasounds still lack the ability to perform transvaginal imaging. Regardless, detecting the size of a normal ovary is of limited clinical value in this context and these device had high diagnostic potential for detecting adnexal abnormalities, as well as none of the sterilization issues of transvaginal assessment, which outweighs this limitation for clinical application in a low-resource setting. Our study differs from prior research in that it includes comprehensive comparison of quantitative measurements of gynecologic structures and calculated sensitivity/specificity, positive predictive value and negative predictive value of this test.

The literature supports the clinical utility of ultrasound testing in a variety of low resource geographic locations and for a broad number of clinical applications with a small number of studies investigating portable POC-US and few investigating handheld devices in low resource settings [5, 7]. The World Health Organization (WHO) endorse the ASSURED guidelines (“affordable, sensitive, specific, user-friendly, rapid and robust, equipment-free and deliverable to end users”) outlining ideal characteristics of a point-of-care (POC) test for use in low-resource settings [22]. These guidelines were developed for laboratory-based POC tests, therefore revised criteria that incorporate clinical considerations for broadened application have been suggested. The ideal clinical POC test is one that allows expeditious clinical decision making, is capable of use at the clinical point of care by health care workers, is affordable, rapid, cost effective and with acceptable test efficacy [23]. The POC-US selected in the current study fulfills these criteria, making it well suited for use in a low resource setting.

In order to better formalize and standardize global research of emerging POC tests for use in a low-resource setting, Drain et al. propose a three-fold assessment of test accuracy, clinical impact, and cost analysis [23]. In the current study, evaluation of POC-US test accuracy as outlined by these authors was successfully achieved. In a pilot study of clinical impact in a low resource setting (Borge, Haiti), we demonstrated feasibility and acceptability of this technology to providers and patients. To our knowledge, this is the first study to examine handheld POC-US performed by gynecologists with gynecologic application in a low resource setting. Future studies are needed to further investigate clinical impact and cost analysis of POC-US for gynecologic application. This higher level of evidence is imperative to inform stakeholders and policy makers.

This study has several limitations. In field study pilot, there was an overall low prevalence of abnormal pelvic ultrasound findings (10%) and no clinically significant pathology identified by POC-US. Authors suspect this low prevalence of pathology was related to relaxed eligibility criteria for enrollment. Any subject with a chief complaint of abdominal pain was enrolled in the study due to potential for gynecologic pathology as an etiology, however stricter inclusion/exclusion criteria for enrollment would likely have resulted in an enriched study population with higher prevalence of gynecologic-specific abdominal pain and gynecologic pathology. This was not possible in the current study due to limitations in sample size and time constraints.

Further limitations are also noted in the validation study portion. The validation study was performed at a single site and therefore results may not be applicable to all clinical settings. Furthermore, validation was performed on a study population with very different demographic characteristics as compared to the field study population, which may limit generalizability and intended application of the results to this and future field studies of POC-US in low resource settings. POC-US images were obtained by a senior resident in Obstetrics and Gynecology and not a board-certified Gynecologist which may limit interpretation for diagnosis. Because this is a pilot study, data is limited by small sample sizes and a sequential convenience sampling strategy. Blinding of the reviewers scoring the ultrasounds for image quality and correlation was achieved by removing all identifying brand information from the images, however blinding was limited by inherent differences in pixel widths for each device that could influence reviewers and their scoring. Although all three devices were rotated in sequence after each subject during image acquisition, this does not eliminate the potential that the operator may have become more familiar or comfortable with one device which would modify the results of comparison testing.

Strengths of the study are that a single clinician performed all POC-US scans, thereby limiting variation among operators, a common limitation of ultrasound technology. Additionally, during validation, all subjects who underwent POC-US also underwent reference imaging with Dx-Us and this crossover methodology allowed subjects to act as their own controls. Clinician was blinded to clinical history and any pathology prior to ultrasound scan, which limited observer bias during bedside image interpretation and diagnosis.

## Conclusion

In summary, clinician performed POC-US is a promising tool that has feasibility and diagnostic accuracy to assess gynecologic anatomy and pathology. In its application to a low resource setting, the devices tested possess characteristics of an ideal POC test as defined by prior authors and the WHO [22, 23]. The device utilized (Phillips Lumify) was small, lightweight and portable, affordable, allowed rapid testing, did not require internet connection or electricity, had 4.5 h of continuous scan time on a single battery charge and the ability to be recharged by solar power or car battery. No infrastructure and only limited maintenance is needed for this device. Through standardized training modules, there is potential for mid-level providers to use these ultrasound devices for immediate diagnosis or to transmit images for remote interpretation. These devices have the potential to be a cost-effective solution to improve diagnosis and management of routine gynecologic pathology worldwide and impact global women’s health care in a meaningful way.

### Device sources and industry involvement (conflict reporting)

The Vscan (GE) ultrasound equipment was previously purchased by the institution and used on temporary loan from the Department of Obstetrics and Gynecology at University of Rochester for the study period. Sonosite supplied Iviz equipment on temporary loan for the study period at the request of one author (MT). Lumify equipment was rented from Philips for the study period. The authors and University report no conflicts of interest regarding this equipment. None of the authors are or were in any contractual agreement with GE, Sonosite, Philips, Samsung regarding the study equipment (other than purchasing/rental/loan agreements) and none serve as speakers/experts for any of the companies. Neither the authors nor the University received any payment, support, or benefits in relation to this study from these companies (other than equipment loan as noted above). GE, Sonosite, Philips, and Samsung did not have any involvement in development of this study, the analysis or review of the data, writing of the manuscript, and did not have any approval or decision making in the submission of this manuscript.

## Supplementary information

**Additional file 1. Table S1**: Evaluating the accuracy of POC-US for gynecologic pathology.

## Data Availability

The datasets used and/or analyzed during the current study are available from the corresponding author on reasonable request.

## Appendix 1

GE Vscan 1.2 (GE Vingmed Ultrasound, Horten, Norway) is a 390 g handheld ultrasound measuring 135 × 73 × 28 mm with a 1.7–3.8 MHz single sector phase array probe and a docking station for recharging. The display screen has resolution of 240 × 320 pixels and is 3.5 inches. The device has capability for color Doppler flow and basic measurements. Continuous scan time on one battery charge is 60–75 min and captured images are saved onto micro-SD card of the device with 32 GB of storage and can be later transferred to computer via microUSB cable. The estimated cost of this unit is around $3,300.00 with a 3-year warranty.

Iviz (FUJIFILM Sonosite, Washington, DC, USA) is a 520 g handheld ultrasound with overall dimensions 18.3 × 11.7 × 2.7 cm, display screen 17.8 cm with 1290 × 1200 pixel resolution and a tablet-like touchscreen interface. The device has 2D, M-mode, Color Doppler and THI capabilities. Continuous scan time on one battery charge is 60 min, though this depends on use of additional features such as the color box. The unit comes with 3 rechargeable batteries that may be interchanged to increase scanning time in the field. A 5–1 MHz phased probe with scan depth 32 cm and 10–5 MHz linear probe with scan depth 9 cm may be selected. Captured images are saved directly onto device which has 64 GB of storage (potential to store 250,000 images or 4000 4-s clips). Images can later be transferred wirelessly to a computer via internet connection. The estimated cost of a unit is $9,500.00 and includes a 3-year warranty.

Philips Lumify (Koninklijke Philips B.V, Amsterdam, Netherlands) is an app-based ultrasound probe compatible with a variety of android-based handheld mobile devices and tablets. Available probes include 4–1 MHz sector array, 5–2 MHz curved array, and 12–4 MHz linear array. In the current study, the 5–2 curved array was used in combination with Samsung Galaxy Tab S2 9.7.

This system weighs 389 g and has a display measuring 9.7 inches and pixel resolution 2048 × 1536. Continuous scan time per battery charge is 4 h 30 min (based on capabilities of the chosen tablet) with battery charging time of 4.68 h. Captured images are saved onto the device which has 32 GB of storage. Images can later be transferred wirelessly via internet connection or via microUSB cable to computer. The cost of this unit is approximately $4326.00 ($3,993.06 for the Lumify curvilinear array and $332.44 for the chosen tablet) with a 12-month warranty.

**Table:**

	Vscan	Iviz	Lumify
Battery charging time	90 min	120 min using external charger 4 h with battery in the system using USB charger	Depends on tablet device used
Presets	Cardiac, Abdominal Obstetrics	Depends on probe selected	Gallbladder, Abdomen, Lung, OBGYN
Depth Adjustment	6–24 cm	5.1–32 cm	1–30 cm
Gain Adjustment	No, Autogain	Yes (0–100); near gain and far gain present	Yes (0–100)
Color Flow	Yes	Yes	Yes, fast flow and slow flow
M-mode	No	Yes	Yes
Cine	No	Yes, 256 frames	No
Save loop	Yes (2 s)	Yes (2, 4, 6, 10, 15, 30 and 60 s)	Yes (3–10 s)
Power	No	No	Yes (0 to negative 30)
Dynamic range	No	Yes	No
Measurements	Caliper distance (single per image)	Caliper distance and volume (multiple per image)	Caliper distance and volume (multiple per image
	No additional calculation packages	Additional calculation packages	Additional calculation packages
Annotatation	Voice notations	Keyboard	Keyboard
		Can't annotate and measure on same image	Can annotate and measure on same image
		Can customize labels	–
Export	USB, Email	Local PACS, Cloud, USB, Email, Printer	DICOM, network share, Local directory, USB, Email
Barcode scanner	No	Yes	Yes
Worklist	No	Yes	Yes
Start-up time	20 s	< 35 s	< 5 s
Controls	Buttons	Touchscreen	Touchscreen
Ability to reopen a closed study	No	No	No
Structured reports	No	Yes	Yes

## Abbreviations

POC-US: Point of care ultrasound
Dx-Us: Diagnostic ultrasound
Vscan: GE Vscan point of care handheld ultrasound device
Iviz: Sonosite Iviz point of care handheld ultrasound device
Lumify: Phillips Lumify point of care handheld ultrasound device
GYN: Gynecologic
PPV: Positive predictive value
NPV: Negative predictive value
RDMS: Registered diagnostic medical sonographer
OB/GYN: Obstetrics and gynecology
FDA: Food and Drug Administration
WHO: World Health Organization
POC: Point-of-care
