# Supplementary material for: Evaluating sensitivity and specificity of handheld point-of-care ultrasound testing for gynecologic pathology: a pilot study for use in low resource settings
Source: BMC Med Imaging. 2020 Oct 27;20:121. doi: 10.1186/s12880-020-00518-8 (PMC7590494; doi:10.1186/s12880-020-00518-8)
Supplement: Supplementary file 1 — Additional file 1. Table S1: Evaluating the accuracy of POC-US for gynecologic pathology. [file 12880_2020_518_MOESM1_ESM.pdf]

Appendix B

Supplemental table 1. Evaluating the accuracy of POC-US for gynecologic pathology

			DX-US		
			Pathology present	Pathology absent	Total
Endometrial pathology	POC-US	Pathology present	10	1	11
		Pathology absent	0	27	27
		Total	10	28	38
Adnexal pathology	POC-US	Pathology present	13	4	17
		Pathology absent	4	19	23
		Total	17	23	40
Myoma	POC-US	Pathology present	8	2	10
		Pathology absent	2	27	29
		Total	10	29	39
